# Re-sequencing and optical mapping reveals misassemblies and real inversions on *Corynebacterium pseudotuberculosis* genomes

**DOI:** 10.1038/s41598-019-52695-4

**Published:** 2019-11-08

**Authors:** Thiago de Jesus Sousa, Doglas Parise, Rodrigo Profeta, Mariana Teixeira Dornelles Parise, Anne Cybelle Pinto Gomide, Rodrigo Bentos Kato, Felipe Luiz Pereira, Henrique Cesar Pereira Figueiredo, Rommel Ramos, Bertram Brenig, Artur Luiz da Costa da Silva, Preetam Ghosh, Debmalya Barh, Aristóteles Góes-Neto, Vasco Azevedo

**Affiliations:** 1 Institute of Biological Sciences, Federal University of Minas Gerais, Belo Horizonte, Minas Gerais Brazil; 2 National Reference Laboratory for Aquatic Animal Diseases (AQUACEN) of Ministry of Agriculture, Livestock and Food Supply, Federal University of Minas Gerais, Belo Horizonte, Minas Gerais Brazil; 3 Institute of Biological Sciences, Federal University of Pará, Belém, Pará Brazil; 4 Institute of Veterinary Medicine, University Göttingen, Göttingen, Germany; 5 Department of Computer Science, Virginia Commonwealth University, Richmond, United States; 6 Institute of Integrative Omics and Applied Biotechnology, Nonakuri West Bengal, India

**Keywords:** Genome assembly algorithms, Imaging and sensing

## Abstract

The number of draft genomes deposited in Genbank from the National Center for Biotechnology Information (NCBI) is higher than the complete ones. Draft genomes are assemblies that contain fragments of misassembled regions (gaps). Such draft genomes present a hindrance to the complete understanding of the biology and evolution of the organism since they lack genomic information. To overcome this problem, strategies to improve the assembly process are developed continuously. Also, the greatest challenge to the assembly progress is the presence of repetitive DNA regions. This article highlights the use of optical mapping, to detect and correct assembly errors in *Corynebacterium pseudotuberculosis*. We also demonstrate that choosing a reference genome should be done with caution to avoid assembly errors and loss of genetic information.

## Introduction

Next Generation Sequencing (NGS) platforms provide an exponential increase in the amount of data produced in a single assay (high-throughput data). This approach provided the scientific community with the ability to sequence more genomes at reduced costs. The NGS platforms perform the sequencing through different technologies, which were developed by different companies, such as 454 GS FLX system (Roche)^1^; Hiseq paired-end (Illumina)^2^; Ion Torrent PGM (Life Technologies)^3^; PacBio sequel system(Pacific Biosciences); and MinION (Oxford Nanopore)^4^. From these, thousands of genomic projects were created to sequence Bacteria, Archaea, and Eukarya species, viruses, and metagenomes^5^.

The main database of these sequences is GenBank maintained by the National Center for Biotechnology Information (NCBI), which in September 2018, contained 153,992 bacterial genomes, most of these being drafts, and only 11,103 sequences (7%) were complete genome sequences. Furthermore, the complete sequences still might have misassemblies due to the presence of repetitive regions, such as ribosomal RNA (rRNA), transposases, phage regions, and plasmids^6^. These errors bias future studies and inferences, such as in comparative genomic or structural genomic analyses, and even ordering of phylogenetically related genomes^7^. Thus, obtaining a more precise and accurate complete genome sequence of an organism is fundamental to understanding its biological and evolutionary characteristics^7^.

The assembly problem persists even with the increase in the reads size, sequencing quality, and updates of *de novo* assembly algorithms. Another limiting factor to the increase of complete sequences is the lack of trained professionals. However, approaches to support this process have been gaining prominence^8^. For example, the use of SSPACE^9^ software to use paired-end reads to create a consensus sequence and perform scaffolding of contigs. Similarly, MapRepeat^10^ and riboSeed^11^ try to solve the repetitive region’s problem.

In order to solve this assembly problem and to improve the generated data, we have the strategy of optical mapping, or Whole Genome Mapping (WGM), which is an approach that uses high-resolution restriction maps to generate the actual orientation of the organism’s genome. It is the main method of large-scale genome analysis that provides complete visualization of the structural genome through a single image^12^. Optical mapping is based on the distance of the restriction sites for high precision map construction. This is a strategy in which data are obtained with greater precision since it is a physical result of the genome evaluation. This method, combined with the application of *de novo* assembly methodology, assists in the orientation of contigs^13^.

The technique of optical mapping was first developed by Schwartz and collaborators in 1993, with the purpose of studying the chromosomal gene ordering of *Saccharomyces cerevisiae*^13^. Samad *et al*., 1995, describe optical mapping as the novel approach for single-molecule DNA analysis using flowering microscopy to identify and estimate its size by the generated images^14^. Since then, several improvements have been added to the technique, especially in the images and algorithms for fragment size estimation^15^. Hence, WGM gained notoriety in several applications, such as in lineage typing in epidemic cases for clinical microbiology^16,17^; ordering of contigs generated by *de novo* assembly^7^; and in the study of inversions, insertions, deletions, duplications, and instability of bacterial genomes^18,19^. WGM has been successfully performed on very different types of organisms such as bacteria^20–22^, fungi^23^, plants^24–26^, and mammals^27^.

Regarding the genomic assembly strategy, WGM is an additional method that allows the ordering of the contigs and thus provides a size estimation of the gaps and their positions. This combination of methods is called a hybrid approach to scaffolding assembly, and it is feasible to acquire a complete genome of high quality and accuracy^7^.

The work carried out by Mariano and collaborators in 2016, updated the genome of *Corynebacterium pseudotuberculosis* 1002^28^ (later deposited as Cp1002B) using the optical mapping technique. *C*. *pseudotuberculosis* 1002B was the first organism of the *Corynebacterium* genus that had optical mapping applied in the detection and correction of assembly errors. From the results obtained by Mariano and collaborators in 2016, we decided to investigate another 10 genomes with this strategy, namely: *C*. *pseudotuberculosis* 29156 (Cp29156), *C*. *pseudotuberculosis* I19 (CpI19), *C*. *pseudotuberculosis* FRC41 (CpFRC41), *C*. *pseudotuberculosis* T1 (CpT1), *C*. *pseudotuberculosis* 31 (Cp31), *C*. *pseudotuberculosis* Cp162 (Cp162), *C*. *pseudotuberculosis* MB302 (CpMB302), *C*. *pseudotuberculosis* CIP52.97 (CpCIP52.97), *C*. *pseudotuberculosis* MB1 (CpMB11) and *C*. *pseudotuberculosis* 258 (Cp258) (Table 1). Among these genomes, 4 are strains from the biovar *ovis* and six from the biovar *equi*^29^.

**Table 1:** Information on sequencing and assembling of strains.

Strains	Sequencing	Reads	Assembly Software	Length (Mb)	Mapped reads (%)	Accession number	Reference
1002B	Ion PGM 200 bp	739,755	Mira v. 3.9.18	2.33511	99.70	CP012837.1	Mariano *et al*.^54^
29156	Ion PGM 200 bp	1,400,026	Newbler v. 2.9	2.33865	98.02	CP010795.1	On this work
I19	Ion PGM 400 bp	1,255,111	Spades v. 3.6.0	2.33759	99.64	CP002251.2	On this work
31	Ion PGM 400 bp	1,394,211	SPAdes 3.6.0	2.40296	99.57	CP003421.3	Viana *et al*.^55^
162	Ion PGM 200 bp	2,050,404	Newbler v. 2.9.	2.36587	98.00	CP003652.2	On this work
258	Ion PGM 200 bp	260,169	Spades v. 3.6.0	2.36982	99.41	CP003540.2	Mariano *et al*.^56^
CIP52.97	Ion PGM 400 bp	1,427,084	Mira v. 3.9.18	2.36939	99.68	CP003061.2	On this work
MB302	Ion PGM 400 bp	1,832,580	Newbler v. 2.9	2.36881	99.59	CP021982.1	Baraúna *et al*.^57^
T1	Ion PGM 200 bp	1,118,022	Newbler v. 2.9	2.3372	95.93	CP015100.1	Almeida *et al*.^58^
MB11	Ion PGM 200 bp	6,753,458	Mira 4.0.2	2.36342	99.24	CP013260.1	Baraúna *et al*.^59^

The strategy is to make *C*. *pseudotuberculosis* the most used organism in genomic studies involving the *Corynebacterium* genus. Therefore, a total of 11 strains were selected from different hosts, isolation sites and distributed between *ovis* and *equi* biovars, so that complete genomes can be made available, well assembled, and updated by new sequencing. In this manner, this data can be explored with greater reliability by future comparative studies, intraspecific evolutionary relationship analyses.

## Results

### Sequencing and assembly

The strains deposited by our research group were either re-sequenced (i.e., Cp1002 (1002B), CpI19, Cp31, Cp162, Cp258, CpCIP52.97), or were first sequenced using the Ion Torrent PGM^TM^ platform (i.e., Cp29156, CpMB302, CpMB11, CpT1) (Table 1). Different software packages were used for *de novo* assembly (Table 1).

### Optical mapping analysis: biovar *ovis* strains

**Figure 1:**
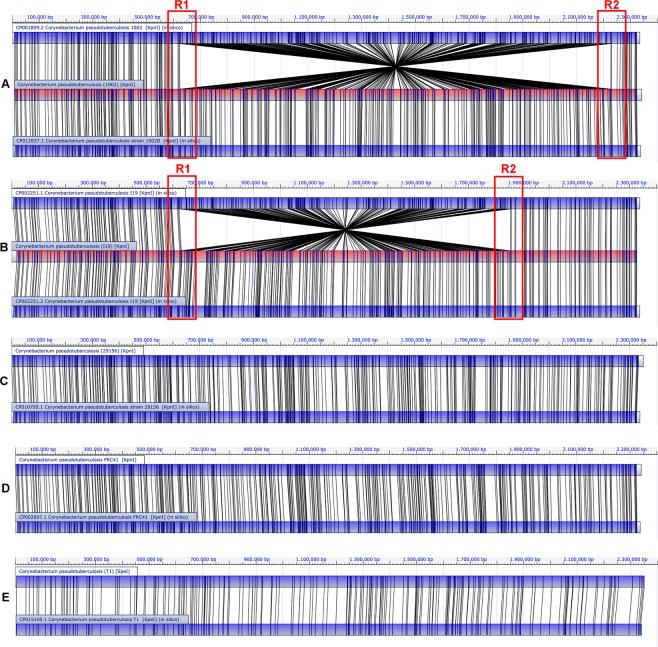
Optical map alignment of the selected *ovis* biovar strains. Comparisons between the first and the new version (when available), with *C. pseudotuberculosis* 1002 and 1002B (**A**); *C. pseudotuberculosis* I19 (**B**); *C. pseudotuberculosis* 29156 (**C**); *C. pseudotuberculosis* FRC41 (**D**); *C. pseudotuberculosis* T1 (**E**) are shown. R1 and R2 highlighted regions are events of inversion errors.

The strains Cp1002 (CP001809.2) and CpI19 (CP002251.1) (Fig. 1A,B) showed an inversion of approximately 1.6 Mb and 1.22 Mb, respectively. It is observed in the regions flanking the first and third clusters of Ribosomal RNA in the CpI19 strain; while in Cp1002, it occurs between the first and fourth clusters. Figure 1A,B show the starting and ending points of the inversion, labeled as R1 and R2, respectively. The central block in Fig. 1 corresponds to the physical restriction map, while the upper and lower blocks represent the *in silico* restriction map generated by MapSolver^TM^ software. The red regions of the central block (Fig. 1A,B) indicate that the same region exists in both the first version (Upper Block) and the updated version (Lower Block). Thus, they show that there was no significant loss between the compared versions in that region.

Strain CpFRC41 (CP002097.1) (Fig. 1D) showed a correct alignment of the restriction sites in the whole genome and thus, with no probable errors of assembly and ordering. Strains Cp29156 and CpT1 (Fig. 1C,E) were first sequenced in this work.

### Optical mapping analysis: biovar *equi* strains

**Figure 2:**
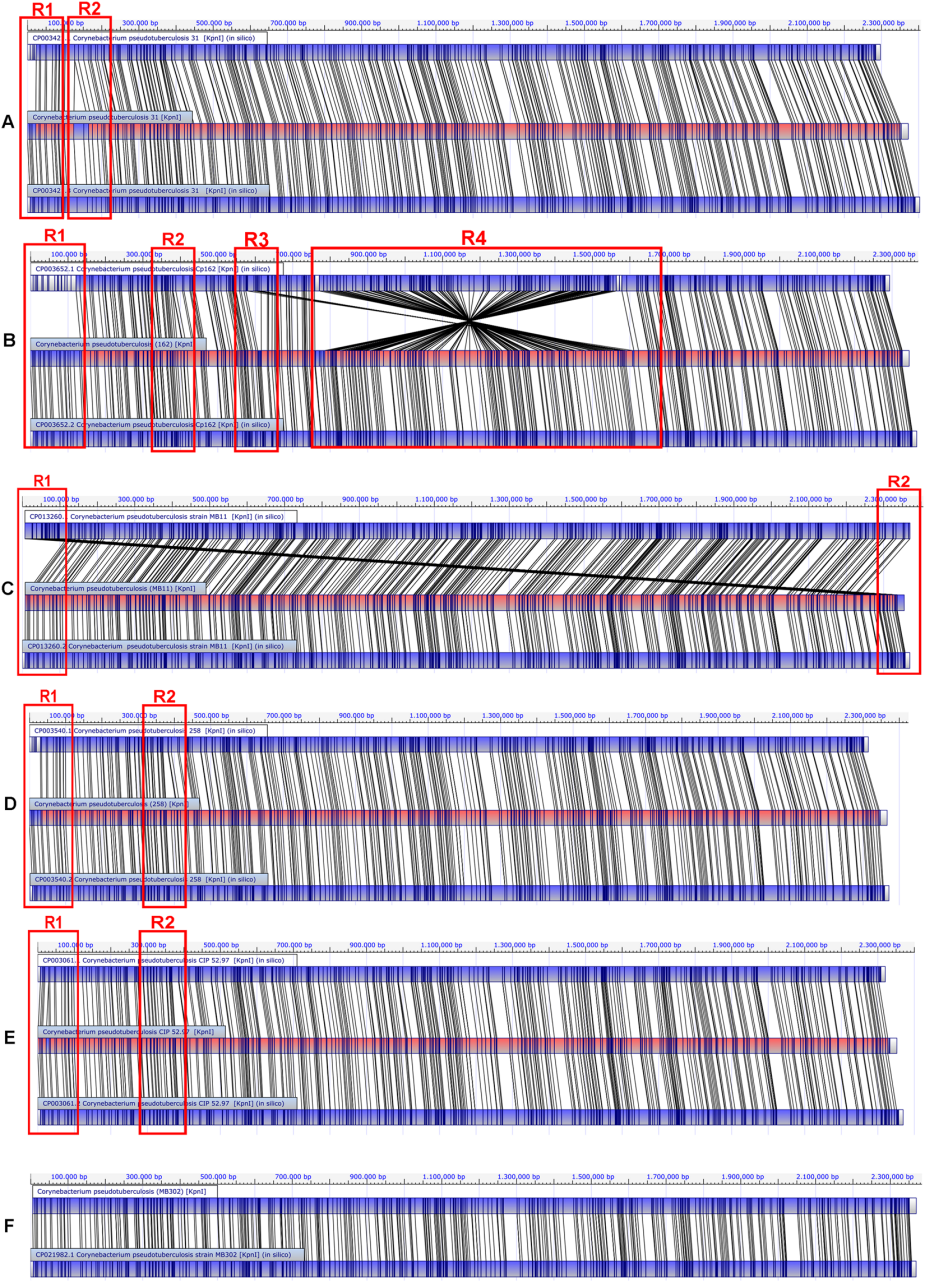
Optical map alignment of the selected *equi* biovar strains. Comparisons between the first and the new version (when available), with *C. pseudotuberculosis* 31 (**A**); *C. pseudotuberculosis* Cp162 (**B**); *C. pseudotuberculosis* MB11 (**C**); *C. pseudotuberculosis* 258 (**D**); *C. pseudotuberculosis* CIP52.97 (**E**); *C. pseudotuberculosis* 302 (**F**) are shown.

Figure 2A shows the alignment between the first (upper map) and the last (lower map) versions of the CpCp31 strain. The R1 region highlights the absence of corresponding restriction sites at the beginning of the genome. R2 region, in its turn, shows the absence of a chromosome region. This difference probably occurred due to errors in the assembly and gap closure process.

Worse problems were found in the previous version of Cp162 (Fig. 2B). The R1-labeled region, starting in 5′ end of the *dnaA* gene, shows no similarity between the restriction site patterns. In the R2 region, there is no linear alignment between the sites, probably due to the absence of genes. According to the optical map, an error may have occurred in the ordering of contigs in the R3 region, where the segment should be in another region of the genome. R4 region shows a ~0.85 Mb inversion in the middle of the genome, located explicitly between two clusters of ribosomal RNA.

The CpMB11 strain presented an error of choice of chromosome initiation site, in which a segment close to 5′ end of the *dnaA* gene should be situated at the end (Fig. 2C, regions R1 and R2). The Cp258 strain did not present chromosomal inversions in the deposited genome. However, a misalignment of the restriction sites (Fig. 2D, region R1) is shown next to the origin of replication at the 5′ end of the genome. Also, a region containing the genes *moaE*, *molB*, *molA*, *narI*, *narJ*, *narH*, *narG*, *narK*, *narT*, *moeY*, *moaC* is absent in the deposited chromosome (Fig. 2D, R2). The same situation occurred with the strain CpCIP5297 (Fig. 2E, regions R1, and R2). Finally, as for the Cp29156 and CpT1 strains, it is the first sequencing of the CpMB302 strain (Fig. 2F).

### Content and genomes plasticity

**Figure 3:**
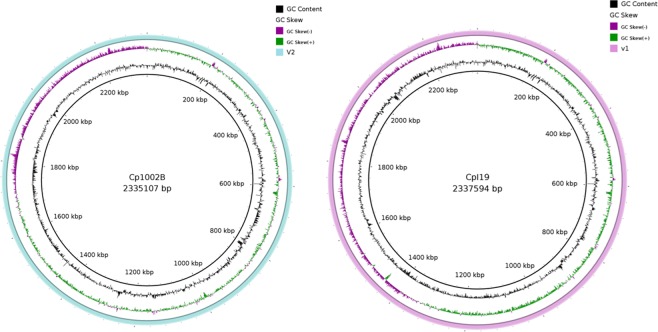
Comparative BRIG analysis of *ovis* biovar strains. Comparative genomic maps of the older versions (outermost circles) and their respective versions with optical map (inner black circles). (**A**) *C. pseudotuberculosis* 1002B. (**B**) *C. pseudotuberculosis* I19.

This analysis showed a reduction of 136 bp in the total genome of the CpI19 strain, in addition to an increase of 34 CDSs and a reduction of 12 pseudogenes (Table 2). The updated version of strain Cp1002^28^ showed a reduction of 6 bases in the total genome and a reduction in the number of annotated CDSs (n = 24) when compared with the older one. In this case, updating the annotation of genes identified as hypothetical protein might be the explanation. This comparison can be visualized in the map generated by BRIG, in which the last version is compared with the most updated one before the optical mapping. Strains Cp1002B (Fig. 3A) and CpI19 (Fig. 3B) showed no gaps in the comparison, meaning that there were no relevant losses or gains among the versions.

**Table 2:** Comparison between deposited and new version assembly of CpI19, Cp1002 (Cp1002B), Cp258, Cp162, Cp31, and CpCIP52.97 strains.

Isolates	Bases (bp)	CDS	Pseudogenes
I19^1^^st^	2,337,730	2,095	57
I19^2^^nd^	2,337,594	2,129	45
1002^2nd^	2,335,113	2,095	47
1002B^1^^st^	2,335,107	2,071	43
258^1^^st^	2,314,404	2,088	46
258^2^^nd^	2,369,817	2,129	34
162^1^^st^	2,293,464	2,002	87
162^2^^nd^	2,365,874	2,099	43
31^1^^st^	2,297,010	2,063	46
31^3rd^	2,402,956	2,173	4
CIP52.97^1^^st^	2,320,595	2,060	75
CIP52.97^2^^nd^	2,369,387	2,187	62

**Figure 4:**
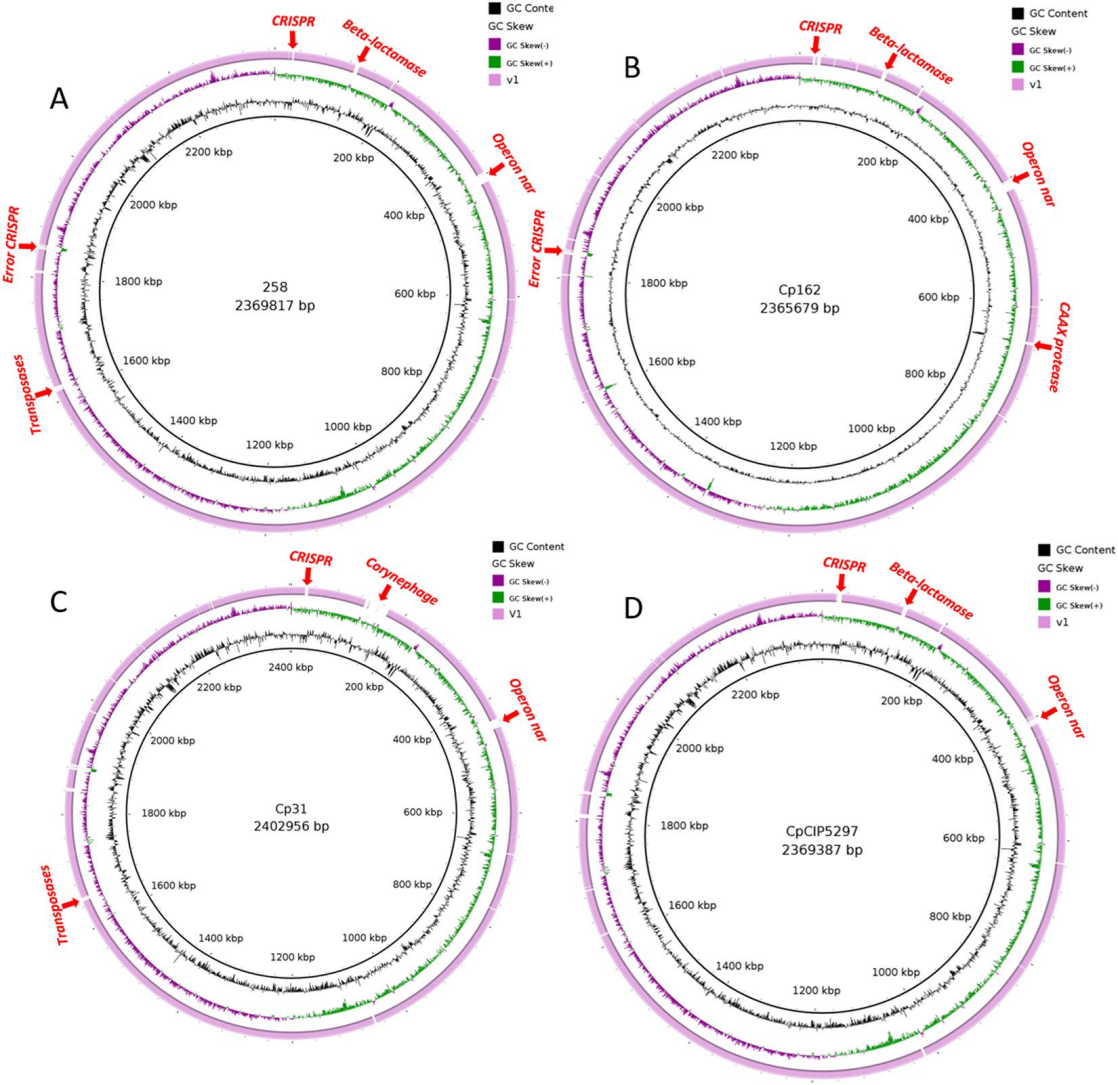
Comparative BRIG analysis of equi biovar strains. Comparative genomic maps of the older versions (outermost circles in purple) and their respective versions with optical map (inner black circles). (**A**) *C. pseudotuberculosis* 258. (**B**) *C. pseudotuberculosis* Cp162. (**C**) *C. pseudotuberculosis* 31. (**D**) *C. pseudotuberculosis* CIP52.97.

The *equi* strains showed more changes among the genomes. In the Cp258 genome, obtained from the Ion Torrent PGM sequencing, an insert of ~55 kb was added. A total of 41 new CDSs and a reduction of 12 pseudogenes were included (Table 2). This had not been represented within the first genome, which was obtained by SOLiD v3 sequencing. Differences can also be visualized on the genomic map (Fig. 4A), where the highlighted red genes are important and essential genes for the strain classification e.g., the operon *nar*, with the *moaE*, *moaD*, *molB*, *molA*, *narI*, *narL*, *narG*, *narK*, *narT*, *moEY*, *mobA*, *moAc*, *moeA1* genes. Presence of genes coding for proteins such as Beta-lactamase, Vitamin K-dependent gamma-carboxylase, Heavy-metal-binding protein, Transposases, Type I restriction-modification system, and N-6 DNA Methylase is also important. Moreover, errors related to positioning and presence of CRISPR associated proteins were found among the assemblies.

The strain Cp162 also presented an increase of genomic content (~72 kb). Ninety-seven CDS and a reduction of 44 pseudogenes were found (Table 2). Figure 4B shows the absent regions of genes such as the complete cluster of the operon *nar*. Genes coding for Fe^3+^ dicitrate transport, ATP-binding protein FecE, Beta-lactamase, Vitamin K-dependent, gamma-carboxylase, Heavy-metal-binding protein, Phytoene dehydrogenase, CAAX protease self-immunity, Restriction endonuclease or methylase, Collagen-binding surface protein Cna-like, B-type domain protein, Membrane protein, ATP-dependent exonuclease, and several hypothetical proteins were also absent. A possible assembly error in the cluster of genes coding for CRISPR-associated proteins was found.

The new sequencing by Ion Torrent of the strain Cp31 resulted in the most significant increase in the gene content (~106 kb) among the selected strains. An increase of 110 CDSs and a reduction of 42 pseudogenes (Table 2) were detected. In Fig. 4C, we can highlight the inclusion of the corynephage with the tox gene of diphtheria toxin.

The CpCIP52.97 strain had an increase of ~49 kb, which represented a gain of 127 CDSs and the reduction of 13 pseudogenes (Table 2). In Fig. 4D, we can highlight the absence of essential genes, such as genes associated with CRISP (*cas2*, *cas1*, *cas3*, *cas4*, *cas5*, *cas6*, *cas7*). Once more, the operon *nar* was absent, as well as genes coding for Beta-lactamase, Phytoene dehydrogenase, integrins, transposases, and several hypothetical proteins.

**Figure 5:**
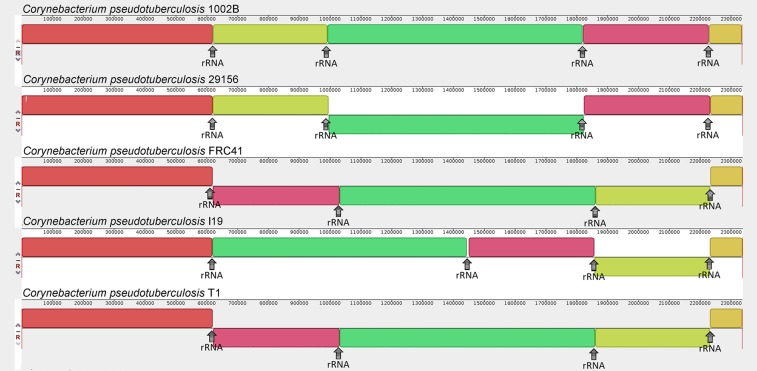
Comparative MAUVE analysis of *ovis* biovar strains. Comparison of Genome alignment of *C. pseudotuberculosis* 1002B, *C. pseudotuberculosis* 29156, *C. pseudotuberculosis* FRC41, *C. pseudotuberculosis* I19 and *C. pseudotuberculosis* T1 strains.

**Figure 6:**
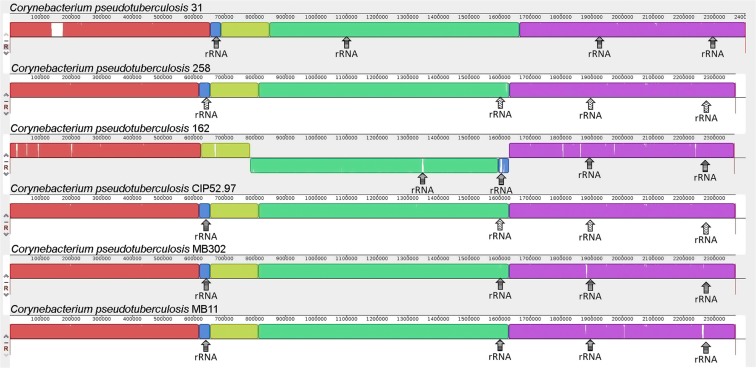
Comparative MAUVE analysis of *equi* biovar strains. Comparison of Genome alignment of *C. pseudotuberculosis* 31, *C. pseudotuberculosis* 258, *C. pseudotuberculosis* Cp162, *C. pseudotuberculosis* CIP52.97, *C. pseudotuberculosis* MB302 and *C. pseudotuberculosis* MB11.

With the complete and finalized genomes, a multiple alignment analysis of the genome was done using Mauve. Figure 5 shows the genomes of 5 *ovis* strains (i.e., Cp1002B, Cp29156, CpFRC41, CpI19, and CpT1). Blocks with the same color represent conserved regions, in which they share a high similarity. Inversions and rearrangement events were established as changes in the Cp1002B reference synteny (first genome of the figure). By analyzing the ends of the inverted blocks, the inversions are flanked by clusters of rRNAs. Only the CpFRC41 and CpT1 strains showed the same gene order (synteny) in their genome. Using the same strategy to compare the *equi* strains (e.g., Cp31, Cp258, Cp162, CpCIP52.97, CpMB302, and MB11), the strain Cp162 was the only one that showed an inversion and rearrangements (Fig. 6). However, these blocks are not flanked by the rRNA clusters.

## Discussion

Although the optical map initially focused on typing and identification of strains without the need of sequencing, it is an efficient approach to order contigs generated by *de novo* assembly, allowing error detection and an accurate contig ordering in assemblies^30^. By definition, the optical mapping technique is a single molecule barcode using restriction enzymes, and the distances among these restriction sites, which are the basis for the alignments between optical maps and the *in-silico* maps (by contigs or genomes). These features are excellent for the scaffolding process with *de novo* assembled contigs^31^. Onmus-Leone and collaborators in 2013 applied this technique to successfully order and correct contigs generated in the *de novo* assembly step using pyrosequencing data of the bacteria *Providencia stuartii*. The mentioned authors established the strategy of using the optical map to order and correct contigs generated from short reads^32^.

Latreille and collaborators in 2007 highlighted the efficiency of the optical map suggesting it as a routine procedure in the assembly finishing process using *de novo* assembly^33^. According to these authors, it is possible to detect errors in the order and construction of the contigs by using this technique even when the organism presents several repeated regions. In the mentioned work, it was possible to finish the assembly by using optical map data even when cosmid libraries and overlapping restriction maps of BACs^33^ have already been applied without success. Analyzing these results, it was possible to conclude that the optical maps are an excellent option for bacterial assembly finishing because the restriction sites cover these repetitive regions in most cases.

Repetitive regions are considered a major difficulty for *de novo* assembly algorithms, mostly in transposons and ribosomal RNA cluster regions^33^. The strain Cp1002 was sequenced by using 454 Genome Sequencer FLX (Roche), Sanger e PacBio technologies, but the error of inversion in rRNA clusters continued. Only when sequencing with Ion Torrent PGM^TM^ and ordering the contigs by using optical mapping data were done, the genome was correctly finished. The strain CpI19 was, at first, sequenced by using SOLiD v2 technology with 25 bp mate-paired reads and a coverage depth of 321-fold. The mate-paired technology may have contributed to the correct construction of the contigs, but due to its small size of reads sequenced, the inversion of the ribosomal RNA cluster regions occurred.

Trost and collaborators sequenced CpFRC41 strain in 2010^34^ by using 454 Genome Sequencer FLX (Roche) sequencer; it was the first *C*. *pseudotuberculosis* strain deposited in GenBank by NCBI in 2010. The assembly was performed using *Corynebacterium diphtheriae* NCTC 13129 (BX248353.1) as the reference genome, and the gaps were closed by using Polymerase Chain Reaction (PCR) and the software r2cat^35^. Gap filling by using PCR probably contributed to no inversions found in the final genome (Fig. 1D). Schröder and collaborators in 2011 successfully used this approach in *Corynebacterium variabile* DSM 44702^36^.

In CpMB11 strain (Fig. 1E) a standardization problem regarding the region of the 5′ end of the *dnaA* gene was found. Several works have shown that the most common origin of replication in bacteria is *oriC* and the first gene is the chromosomal replication initiator protein DnaA (*dnaA*)^37^. Thus, it is used as a standard pattern to linearize bacterial genomes.

Cp31 have been sequenced using several platforms: Solid v2 (CP003421.1), Ion Torrent PGM^TM^ (CP003421.3) and PacBio. This fact confers more reliability to the assembly of this strain, which leads us to consider it as the reference strain in *equi* biovar. In its last version, published by Viana and collaborators in 2017, it was assembled using optical mapping technology. The strains Cp258, Cp162, Cp31 e CpCIP52.97 were initially sequenced in the SOLiD platform; these strains also belong to *equi* biovar and were characterized by using biochemical tests. Those were re-sequenced using Ion Torrent PGM^TM^ platform and novel genomic regions were added to the genome sequence. Essential genes in regions, such as *nar* cluster and clusters associated with CRISPR proteins, which are only present in *equi* biovar, were added. The missing regions may be caused by an error propagation due to reference contig ordering, because of the first *equi* genome available, the Cp258^38^ strain, was assembled using an *ovis* biovar strain as the reference, which does not completely present the referred clusters. The same issue happened to Cp31^39^ isolate.

Similarly, the Cp162^40^ strain presented the same issue because its contigs have been ordered according to *C*. *pseudotuberculosis* 316 (Cp316) (CP003077.1)^41^. Cp316 strain belongs to *equi* biovar and does not present *nar* operon in its former genomic sequence; it was assembled using the *ovis* strain CpFRC41 as a reference genome for contigs ordering. Presumably, the same problem happened in CpCIP52.97^42^, but the genome used as a reference in the assembly process is not described in the article.

Using a complete genome deposited in public databases as a reference to assemble novel genomes is a risky strategy because even if it generates complete genomes more efficiently, it may disseminate assembly errors from one strain to other^8^. In this article, the optical map is used to validate contigs, and it is shown that extension and gap filling using read mapping or *de novo* assembly may generate assembly errors. We highlighted this technique because it does not present sequencing bias. Assembly statistics such as N50, coverage and depth coverage may generate false positive answers. Another strategy suggested by Lehri and collaborators is to use non-paired reads together with paired or long reads. A genomic region presenting assembly errors caused by insertions, deletions, inversions or rearrangements may hide significant biological variations or produce false interpretations, mostly in genomic analysis^43^. Even before the NGS platforms boomed, Schmutz and collaborators (2004) were concerned about the quality of the human genome^43^, mostly because of the possible assembly errors.

The inversions caused by RNA ribosomal clusters in *ovis* biovar strains may occur due to the high similarity of these clusters. This kind of inversion has already been shown in literature in bacteria as *Salmonella paratyphi* A using restriction enzymes and pulsed-field electrophoresis gel (PFGE)^44^. The inversions among strains of the same species may be comprehended as homologous recombination events^45^. It can be highlighted that these inversions do not occur in strains belonging to *equi* biovar, except for Cp162. The inferred data concerning the genomic order of *C*. *pseudotuberculosis* strains were only achieved because optical mapping technology provides an accurate *in vitro* evidence.

## Methods

### Strain and DNA isolation and Genome sequencing

The methodology described below was applied to the novel sequencings of the strains *C. pseudotuberculosis* 1002(1002B), *C. pseudotuberculosis* 29156, *C. pseudotuberculosis* I19, *C. pseudotuberculosis* Cp162, *C. pseudotuberculosis* 258, *C. pseudotuberculosis* 31, *C. pseudotuberculosis* MB302, *C. pseudotuberculosis* MB11, *C. pseudotuberculosis* T1 and *C. pseudotuberculosis* CIP5297. The strains were cultivated in solid media with 1.5% of bacteriological agar. Subsequently, an isolated colony was used to grow in liquid media with brain-heart-infusion media (BHI-Hi Media Laboratories Pvt. Ltd, India) supplemented with 0.5% of Tween 80, at 37 °C for 20 hours under rotation. Genomic DNA was extracted following the protocol of Pacheco in 2006^46^. After the extraction step, the libraries were constructed with IonXpress™ Plus DNA Fragment Library Preparation Kit. The DNA samples were fragmented using Ion Shear^TM^ Plus for five minutes at 37°. Then, adaptors from Ion Xpress™ Barcode Adapters kit were ligated for library quantification. Subsequently, the fragments were amplified using Ion PGM™ 200 bp or 400 bp kits. These reactions were transferred to the semiconductor chip (ION 318^TM^ Chip v2), and it was put into Ion PGM™. During all the steps described above, all the manufacturer’s instructions were strictly followed. No novel sequencing was performed for *C. pseudotuberculosis* FRC41.

### Genome assembly and annotation

The analysis of the reads quality was performed by using FastQC software (http://www.bioinformatics.babraham.ac.uk/projects/fastqc). No trimming was performed on reads with Phred score above 20, which were the majority. For contigs construction, we applied the *de novo* assembly strategy (no reference used) by using Mira v. 3.9.18^46^, MIRA 4.0.2^46^, Spades v. 3.6.0^47^ e Newbler v. 2.9^48^ (Table 2) software. Scaffolds construction was manually performed in CLC Genomics Workbench (CLC-gw) version 7.0 (Qiagen) software using the visualization of the contigs mapped and ordered according to the restriction sites of the strains in MapSolver^TM^ (OpGen). Then, *dnaA* gene was fixed in the probable *oriC* position in the chromosome by using an in-house python script. In order to fill the gaps and finish the assembly, GapBlaster^49^ and FGAP^50^ software was used and subsequently, the contigs were mapped to the scaffold or a reference genome by using CLC Genomics (Qiagen). The annotation was performed by using in-house scripts for annotation transference from *C. pseudotuberculosis* strains, which were manually curated in the UniProt database (http://uniprot.org). Finally, pseudogenes were manually curated by using Artemis software^51^ and CLC Genomics (Qiagen).

### Optical mapping

The optical maps were acquired from Opgen, Inc. The MapSolver™ (OpGen Inc.) software was used for the comparison of the physical restriction map and the restriction sites present in the assembled genome. Several pieces of information about metrics for the quality of each optical map are available in Table 3.

**Table 3:** Information about the quality metrics of the optical maps used.

Strains	Enzyme	Length (bp)	Number of fragments	Average fragment size (bp)	Maximum fragment size (bp)	Minimum fragment size (bp)	Whole genome coverage
1002B	*Kpn1*	2,335,144	353	6,615.139	38,715	903	99.998%
29156	*Kpn1*	2,351,288	368	6,389.37	38,839	1,275	99.462%
I19	*Kpn1*	2,326,586	333	6,986.745	38,215	1,460	100.473%
FRC41	*Kpn1*	2,341,893	369	6,346.593	38,942	1,394	99.830%
31	*Kpn1*	2,372,071	346	6,855.697	35,806	1,544	101.302%
162	*Kpn1*	2,345,656	362	6,479.713	28,013	1,509	100.861%
258	*Kpn1*	2,366,195	346	6,838.714	36,249	1,667	100.153%
CIP52.97	*Kpn1*	2,352,141	347	6,778.504	36,145	1,567	100.733%
MB302	*Kpn1*	2,363,709	362	6,529.583	36,517	1,471	100.215%
T1	*Spe1*	2,350,532	193	12,178.922	54,138	1,787	99.432%
MB11	*Kpn1*	2,347,572	366	6,414.131	36,155	1,333	100.679%

### Genome plasticity and genetics content

This analysis was performed using genomic sequences before and after assembly assisted by optical mapping data. The maps comparing different versions of the studied strains were generated by using Blast Ring Image Generator (BRIG) v0.95^52^. For inversion, deletion and rearrangement analysis, the Mauve v. 2.3.1^53^ software was used with progressiveMauve^53^ option set.

## Conclusion

The results obtained from optical mapping data analysis pointed errors in the assemblies of *C*. *pseudotuberculosis* genomes deposited in Genbank. Thus, the optical map was efficient in the assembly error detection of the strains Cp1002, CpI19, Cp31, Cp162, CpMB11, Cp258, and CpCIP52.97. Regarding the novel genomes, such as Cp29156, CpT1, and MB302, the optical map data contributed in the contigs ordering step, which contributed to a more efficient assembly finishing considering there are no assembly errors in the final version of the genomes. Furthermore, the update of the genomic sequences done by re-sequencing the genomes with Ion Torrent PGM^TM^ platform was essential to the relevant genomic content increase, which happened in the strains previously sequenced using the SOLiD platform. We also pointed out several inversions caused by ribosomal RNA gene clusters in strains of the *ovis* biovar. Thus, we can suggest that the genomes deposited after applying this strategy made these strains more reliable for novel studies.
